# 1-(6-Methyl-3-phenyl-2-sulfanyl­idene-1,2,3,4-tetra­hydro­pyrimidin-5-yl)ethanone

**DOI:** 10.1107/S1600536811055309

**Published:** 2012-01-07

**Authors:** Emin N. Garibov, Sevinj S. Gojayeva, Mirze A. Allahverdiyev, Atash V. Gurbanov Gurbanov, Iván Brito

**Affiliations:** a Department of Organic Chemistry, Baku State University, Baku, Azerbaijan; b Departamento de Química, Facultad de Ciencias Básicas, Universidad de Antofagasta, Casilla 170, Antofagasta, Chile

## Abstract

In the title compound, C_13_H_14_N_2_OS, four C atoms of the phenyl ring are disordered over two sets of sites in a 0.60 (3):0.40 (3) ratio. The heterocyclic ring is essentially planar (r.m.s. deviation = 0.017 Å) and forms dihedral angles of 82.0 (2) and 79.3 (3)°, respectively, with the major and minor occupancy components of the phenyl ring. The crystal packing features N—H⋯O hydrogen bonds, which link the mol­ecules into *C*(6) chains running parallel to the *b* axis.

## Related literature

For synthetic methods, see: Kotharkar *et al.* (2006 ▶); Lu *et al.* (2000 ▶); Salehi *et al.* (2003 ▶); Srinivas & Das (2004 ▶). For pharmacological properties of related compounds, see: Dalinger *et al.* (2004 ▶). For graph-set notation see: Bernstein *et al.* (1995 ▶).
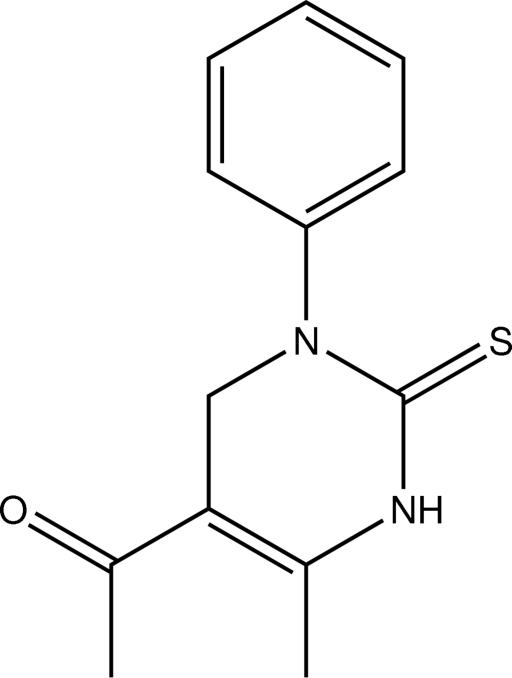


## Experimental

### 

#### Crystal data

C_13_H_14_N_2_OS
*M* *_r_* = 246.32
Orthorhombic, 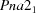

*a* = 24.3527 (10) Å
*b* = 7.2374 (3) Å
*c* = 7.0063 (3) Å
*V* = 1234.86 (9) Å^3^
*Z* = 4
Mo *K*α radiation
μ = 0.25 mm^−1^
*T* = 100 K
0.30 × 0.30 × 0.30 mm

#### Data collection

Bruker APEXII CCD diffractometer
Absorption correction: multi-scan (*SADABS*; Sheldrick, 2003 ▶) *T*_min_ = 0.930, *T*_max_ = 0.930
13745 measured reflections
3089 independent reflections
2877 reflections with *I* > 2σ(*I*)
*R*_int_ = 0.016

#### Refinement

*R*[*F* ^2^ > 2σ(*F* ^2^)] = 0.051
*wR*(*F* ^2^) = 0.141
*S* = 1.00
3089 reflections
150 parameters
19 restraints
H-atom parameters constrained
Δρ_max_ = 0.40 e Å^−3^
Δρ_min_ = −0.35 e Å^−3^

### 

Data collection: *APEX2* (Bruker, 2005 ▶); cell refinement: *SAINT-Plus* (Bruker, 2001 ▶); data reduction: *SAINT-Plus*; program(s) used to solve structure: *SHELXTL* (Sheldrick, 2008 ▶); program(s) used to refine structure: *SHELXTL*; molecular graphics: *SHELXTL*; software used to prepare material for publication: *SHELXTL*.

## Supplementary Material

Crystal structure: contains datablock(s) I, global. DOI: 10.1107/S1600536811055309/lr2042sup1.cif

Structure factors: contains datablock(s) I. DOI: 10.1107/S1600536811055309/lr2042Isup2.hkl

Supplementary material file. DOI: 10.1107/S1600536811055309/lr2042Isup3.cml

Additional supplementary materials:  crystallographic information; 3D view; checkCIF report

## Figures and Tables

**Table 1:** Hydrogen-bond geometry (Å, °)

*D*—H⋯*A*	*D*—H	H⋯*A*	*D*⋯*A*	*D*—H⋯*A*
N3—H3*N*⋯O1^i^	0.88	2.05	2.920 (2)	168

Symmetry code: (i) 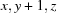.

**Fig. 1.**
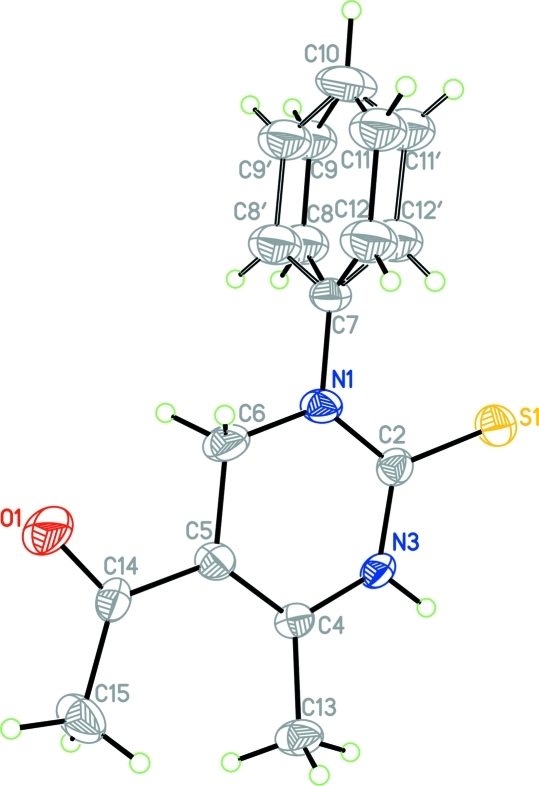
The molecular structure of the title compound, including disorder. Displacement ellipsoids are drawn at the 30% probability level.

**Fig. 2.**
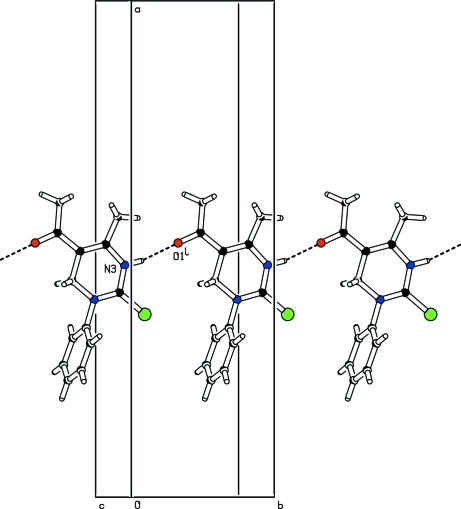
Packing diagram of the title compound. N—H···O hydrogen bonds are shown as dashed lines. For clarity only one of the disordered components of the phenyl ring is shown.

## supplementary crystallographic information

### Comment

The dihydropyrimidinethiones display many pharmacological properties (Dalinger *et al.* 2004), as part of our interest in this kind of materials, we report here the synthesis and crystal structure determination of the title compound. Our synthesis is based in the Bidjinelli reaction, which consists on a three-component condensation of an aldehyde, a methylene active compound and an thiourea derivative in acidic media. This procedure is the most simple and useful for the preparation of 3,4-dihydropyrimidene-2(1*H*) thiones (Kotharkar *et al.* 2006; Lu *et al.* 2000; Srinivas & Das, 2004; Salehi *et al.* 2003).

In the compound, the C8, C9, C11 and C12 atoms of the phenyl ring are disordered over two sets of sites in a 0.60 (3):0.40 (3) ratio. The heterocycle ring is essentially planar (r.m.s.= 0.017 Å) and form a dihedral angle of 82.0 (2)° with the phenyl ring.The crystal packing is stabilized by intermolecular N3—H3N···O1 hydrogen bonds (Table 1), which link the molecules into chains running parallel to the *b* axis (Fig.2), with graph-set notation C(6), (Bernstein *et al.* 1995).

### Experimental

Phenylthiourea, 15.2 g (0.1 mol), 37% water solution of formaldehyde (formaline), 3 g (0.1 mol) and 13 g (0.1 mol) of acetoacetic ester were dissolved in 10 ml of ethanol and then 0.5 ml of trifluoroacetic was added. The mixture was vigorously stirred during 4–5 h at room temperature and then cooled and kept one day at 0°C. The white crystals of 1-(6-methyl-3-phenyl-2-thioxo-1,2,3,4-tetrahydropyrimidin-5-yl) ethanone were filtered and washed with dichloromethane. Suitable crystal for diffraction were obtained by slow evaporation from ethanol. The yield was of 19.2 g (70%). Mp 180°C. *R*_f_ = 0.35. Eluent- ethanol:hexane (5:2). ^1^H NMR (300 MHz, DMSO-d6) δ 1.35 (s, 3H, CH_3_), 6.8–7.1 (m, H, Ar), 7.4 (m, H, Ar), 9.35 (s, 1H, NH). ^13^C NMR (75 MHz, DMSO-d6) δ 24, 29, 37, 51, 86, 117, 122, 129, 132, 141, 151, 179 (C=S), 205 (C=O)

### Refinement

H atoms were placed in calculated position and refined using a riding model, with C—H distances in the range 0.95 — 0.99 Å and N—H distance of 0.88 Å, with *U*_iso_(H) = 1.2*U*_eq_(N,C_methylene _and C_aromatic_) and 1.5*U*_eq_(C_methyl_ ).

### Crystal data

**Table:**

C_13_H_14_N_2_OS	*F*(000) = 520
*M**_r_* = 246.32	*D*_x_ = 1.325 Mg m^−^^3^
Orthorhombic, *P**n**a*2_1_	Mo *K*α radiation, λ = 0.71073 Å
Hall symbol: P 2c -2n	Cell parameters from 7985 reflections
*a* = 24.3527 (10) Å	θ = 2.9–28.3°
*b* = 7.2374 (3) Å	µ = 0.25 mm^−^^1^
*c* = 7.0063 (3) Å	*T* = 100 K
*V* = 1234.86 (9) Å^3^	Cube, colourless
*Z* = 4	0.30 × 0.30 × 0.30 mm

### Data collection

**Table:**

Bruker APEXII CCD diffractometer	3089 independent reflections
Radiation source: fine-focus sealed tube	2877 reflections with *I* > 2σ(*I*)
graphite	*R*_int_ = 0.016
φ and ω scans	θ_max_ = 28.4°, θ_min_ = 1.7°
Absorption correction: multi-scan (*SADABS*; Sheldrick, 2003)	*h* = −32→32
*T*_min_ = 0.930, *T*_max_ = 0.930	*k* = −9→9
13745 measured reflections	*l* = −9→9

### Refinement

**Table:**

Refinement on *F*^2^	Primary atom site location: structure-invariant direct methods
Least-squares matrix: full	Secondary atom site location: difference Fourier map
*R*[*F*^2^ > 2σ(*F*^2^)] = 0.051	Hydrogen site location: difference Fourier map
*wR*(*F*^2^) = 0.141	H-atom parameters constrained
*S* = 1.00	*w* = 1/[σ^2^(*F*_o_^2^) + (0.0713*P*)^2^ + 0.8297*P*] where *P* = (*F*_o_^2^ + 2*F*_c_^2^)/3
3089 reflections	(Δ/σ)_max_ = 0.001
150 parameters	Δρ_max_ = 0.40 e Å^−^^3^
19 restraints	Δρ_min_ = −0.35 e Å^−^^3^

### Special details

**Table:**

Geometry. All e.s.d.'s (except the e.s.d. in the dihedral angle between two l.s. planes) are estimated using the full covariance matrix. The cell e.s.d.'s are taken into account individually in the estimation of e.s.d.'s in distances, angles and torsion angles; correlations between e.s.d.'s in cell parameters are only used when they are defined by crystal symmetry. An approximate (isotropic) treatment of cell e.s.d.'s is used for estimating e.s.d.'s involving l.s. planes.
Refinement. Refinement of *F*^2^ against ALL reflections. The weighted *R*-factor *wR* and goodness of fit *S* are based on *F*^2^, conventional *R*-factors *R* are based on *F*, with *F* set to zero for negative *F*^2^. The threshold expression of *F*^2^ > σ(*F*^2^) is used only for calculating *R*-factors(gt) *etc*. and is not relevant to the choice of reflections for refinement. *R*-factors based on *F*^2^ are statistically about twice as large as those based on *F*, and *R*- factors based on ALL data will be even larger.

### Fractional atomic coordinates and isotropic or equivalent isotropic displacement parameters (Å^2^)

**Table:**

	*x*	*y*	*z*	*U*_iso_*/*U*_eq_	Occ. (<1)
S1	0.36824 (2)	1.18702 (7)	0.3739 (4)	0.04639 (18)	
O1	0.51205 (7)	0.42042 (19)	0.3715 (9)	0.0661 (6)	
N1	0.39774 (6)	0.8325 (2)	0.3725 (6)	0.0341 (3)	
C2	0.41226 (7)	1.0102 (2)	0.3775 (7)	0.0314 (3)	
N3	0.46747 (6)	1.04586 (19)	0.3735 (7)	0.0349 (3)	
H3N	0.4761	1.1644	0.3753	0.042*	
C4	0.50891 (7)	0.9147 (2)	0.3784 (6)	0.0280 (3)	
C5	0.49517 (7)	0.7330 (2)	0.3726 (7)	0.0327 (4)	
C6	0.43611 (8)	0.6775 (2)	0.3775 (11)	0.0512 (6)	
H6A	0.4286	0.5959	0.2672	0.061*	
H6B	0.4294	0.6051	0.4950	0.061*	
C7	0.34084 (7)	0.7781 (2)	0.3747 (3)	0.0413 (4)	
C8	0.3095 (2)	0.7676 (8)	0.2110 (7)	0.0577 (9)	0.60
H8A	0.3243	0.8010	0.0903	0.069*	0.60
C9	0.2556 (3)	0.7066 (7)	0.2280 (7)	0.0577 (9)	0.60
H9A	0.2329	0.6976	0.1182	0.069*	0.60
C8'	0.3160 (4)	0.7165 (12)	0.2081 (8)	0.0577 (9)	0.40
H8B	0.3344	0.7152	0.0885	0.069*	0.40
C9'	0.2620 (4)	0.6568 (13)	0.2298 (9)	0.0577 (9)	0.40
H9B	0.2427	0.6130	0.1210	0.069*	0.40
C10	0.23526 (11)	0.6591 (3)	0.4053 (5)	0.0833 (14)	
H10A	0.1983	0.6178	0.4141	0.100*	
C13	0.56501 (7)	0.9991 (3)	0.3745 (8)	0.0406 (4)	
H13A	0.5881	0.9320	0.2833	0.061*	
H13B	0.5814	0.9916	0.5020	0.061*	
H13C	0.5622	1.1289	0.3358	0.061*	
C14	0.53254 (8)	0.5753 (2)	0.3726 (8)	0.0386 (4)	
C15	0.59377 (9)	0.5914 (3)	0.3708 (10)	0.0538 (6)	
H15A	0.6101	0.4693	0.3909	0.081*	
H15B	0.6055	0.6751	0.4729	0.081*	
H15C	0.6058	0.6406	0.2473	0.081*	
C11	0.2664 (2)	0.6689 (8)	0.5722 (7)	0.0577 (9)	0.60
H11A	0.2516	0.6354	0.6928	0.069*	0.60
C12	0.3200 (3)	0.7300 (7)	0.5526 (6)	0.0577 (9)	0.60
H12A	0.3428	0.7392	0.6622	0.069*	0.60
C11'	0.2596 (4)	0.7202 (12)	0.5726 (11)	0.0577 (9)	0.40
H11B	0.2413	0.7218	0.6924	0.069*	0.40
C12'	0.3133 (4)	0.7788 (13)	0.5473 (9)	0.0577 (9)	0.40
H12B	0.3325	0.8225	0.6563	0.069*	0.40

### Atomic displacement parameters (Å^2^)

**Table:**

	*U*^11^	*U*^22^	*U*^33^	*U*^12^	*U*^13^	*U*^23^
S1	0.0357 (3)	0.0330 (3)	0.0704 (4)	0.00649 (18)	0.0000 (5)	−0.0009 (5)
O1	0.0503 (9)	0.0230 (6)	0.1250 (17)	−0.0015 (6)	−0.014 (2)	0.006 (2)
N1	0.0267 (7)	0.0277 (7)	0.0480 (8)	−0.0031 (5)	0.0033 (14)	0.0073 (15)
C2	0.0321 (8)	0.0271 (7)	0.0351 (8)	0.0008 (6)	−0.0035 (14)	−0.0005 (15)
N3	0.0302 (7)	0.0208 (6)	0.0537 (9)	−0.0025 (5)	−0.0048 (14)	−0.0063 (15)
C4	0.0280 (7)	0.0246 (7)	0.0315 (8)	−0.0019 (6)	−0.0049 (12)	0.0061 (13)
C5	0.0305 (8)	0.0246 (7)	0.0429 (9)	−0.0021 (6)	0.0070 (16)	0.0091 (17)
C6	0.0315 (9)	0.0251 (8)	0.0970 (18)	−0.0036 (7)	−0.010 (2)	0.002 (2)
C7	0.0277 (8)	0.0310 (8)	0.0652 (12)	−0.0041 (7)	0.0077 (18)	0.000 (2)
C8	0.0353 (13)	0.052 (3)	0.0853 (11)	−0.0022 (15)	−0.0014 (10)	0.0077 (14)
C9	0.0353 (13)	0.052 (3)	0.0853 (11)	−0.0022 (15)	−0.0014 (10)	0.0077 (14)
C8'	0.0353 (13)	0.052 (3)	0.0853 (11)	−0.0022 (15)	−0.0014 (10)	0.0077 (14)
C9'	0.0353 (13)	0.052 (3)	0.0853 (11)	−0.0022 (15)	−0.0014 (10)	0.0077 (14)
C10	0.0341 (12)	0.0659 (17)	0.150 (4)	−0.0103 (12)	0.026 (2)	0.002 (3)
C13	0.0316 (8)	0.0301 (8)	0.0601 (12)	−0.0051 (7)	−0.0087 (17)	0.0104 (19)
C14	0.0399 (9)	0.0255 (8)	0.0502 (10)	0.0019 (7)	−0.0041 (17)	−0.0089 (17)
C15	0.0383 (10)	0.0368 (10)	0.0864 (17)	0.0087 (8)	0.012 (2)	0.016 (2)
C11	0.0353 (13)	0.052 (3)	0.0853 (11)	−0.0022 (15)	−0.0014 (10)	0.0077 (14)
C12	0.0353 (13)	0.052 (3)	0.0853 (11)	−0.0022 (15)	−0.0014 (10)	0.0077 (14)
C11'	0.0353 (13)	0.052 (3)	0.0853 (11)	−0.0022 (15)	−0.0014 (10)	0.0077 (14)
C12'	0.0353 (13)	0.052 (3)	0.0853 (11)	−0.0022 (15)	−0.0014 (10)	0.0077 (14)

### Geometric parameters (Å, °)

**Table:**

S1—C2	1.6694 (17)	C9—H9A	0.9500
O1—C14	1.227 (2)	C8'—C9'	1.393 (3)
N1—C2	1.334 (2)	C8'—H8B	0.9500
N1—C7	1.441 (2)	C9'—C10	1.391 (3)
N1—C6	1.460 (2)	C9'—H9B	0.9500
C2—N3	1.369 (2)	C10—C11'	1.386 (3)
N3—C4	1.386 (2)	C10—C11	1.395 (3)
N3—H3N	0.8834	C10—H10A	0.9500
C4—C5	1.357 (2)	C13—H13A	0.9800
C4—C13	1.497 (2)	C13—H13B	0.9800
C5—C14	1.460 (2)	C13—H13C	0.9800
C5—C6	1.494 (2)	C14—C15	1.496 (3)
C6—H6A	0.9900	C15—H15A	0.9800
C6—H6B	0.9900	C15—H15B	0.9800
C7—C8	1.380 (3)	C15—H15C	0.9800
C7—C12'	1.384 (3)	C11—C12	1.386 (3)
C7—C8'	1.389 (3)	C11—H11A	0.9500
C7—C12	1.390 (3)	C12—H12A	0.9500
C8—C9	1.391 (3)	C11'—C12'	1.385 (3)
C8—H8A	0.9500	C11'—H11B	0.9500
C9—C10	1.381 (3)	C12'—H12B	0.9500
			
C2—N1—C7	121.21 (14)	C10—C9'—C8'	122.3 (9)
C2—N1—C6	124.76 (15)	C10—C9'—H9B	118.8
C7—N1—C6	113.90 (14)	C8'—C9'—H9B	118.8
N1—C2—N3	116.18 (15)	C9—C10—C11'	121.9 (6)
N1—C2—S1	124.62 (13)	C9—C10—C9'	16.3 (4)
N3—C2—S1	119.08 (13)	C11'—C10—C9'	123.5 (6)
C2—N3—C4	125.84 (14)	C9—C10—C11	123.1 (4)
C2—N3—H3N	114.6	C11'—C10—C11	16.8 (4)
C4—N3—H3N	119.4	C9'—C10—C11	119.2 (6)
C5—C4—N3	118.90 (15)	C9—C10—H10A	118.4
C5—C4—C13	128.30 (16)	C11'—C10—H10A	116.8
N3—C4—C13	112.62 (14)	C9'—C10—H10A	119.7
C4—C5—C14	127.14 (16)	C11—C10—H10A	118.4
C4—C5—C6	119.80 (16)	C4—C13—H13A	109.5
C14—C5—C6	112.96 (15)	C4—C13—H13B	109.5
N1—C6—C5	114.15 (14)	H13A—C13—H13B	109.5
N1—C6—H6A	108.7	C4—C13—H13C	109.5
C5—C6—H6A	108.7	H13A—C13—H13C	109.5
N1—C6—H6B	108.7	H13B—C13—H13C	109.5
C5—C6—H6B	108.7	O1—C14—C5	117.44 (18)
H6A—C6—H6B	107.6	O1—C14—C15	118.47 (18)
C8—C7—C12'	117.3 (6)	C5—C14—C15	124.08 (16)
C8—C7—C8'	16.7 (4)	C14—C15—H15A	109.5
C12'—C7—C8'	121.6 (6)	C14—C15—H15B	109.5
C8—C7—C12	122.0 (4)	H15A—C15—H15B	109.5
C12'—C7—C12	16.2 (4)	C14—C15—H15C	109.5
C8'—C7—C12	121.0 (6)	H15A—C15—H15C	109.5
C8—C7—N1	122.5 (4)	H15B—C15—H15C	109.5
C12'—C7—N1	118.4 (5)	C12—C11—C10	116.4 (6)
C8'—C7—N1	119.9 (5)	C12—C11—H11A	121.8
C12—C7—N1	115.4 (4)	C10—C11—H11A	121.8
C7—C8—C9	117.9 (6)	C11—C12—C7	120.8 (6)
C7—C8—H8A	121.0	C11—C12—H12A	119.6
C9—C8—H8A	121.0	C7—C12—H12A	119.6
C10—C9—C8	119.7 (6)	C12'—C11'—C10	113.1 (9)
C10—C9—H9A	120.2	C12'—C11'—H11B	123.4
C8—C9—H9A	120.2	C10—C11'—H11B	123.4
C7—C8'—C9'	114.8 (9)	C7—C12'—C11'	124.7 (10)
C7—C8'—H8B	122.6	C7—C12'—H12B	117.7
C9'—C8'—H8B	122.6	C11'—C12'—H12B	117.7
			
C7—N1—C2—N3	−179.6 (3)	C12'—C7—C8'—C9'	0.01 (3)
C6—N1—C2—N3	4.8 (7)	C12—C7—C8'—C9'	19.0 (5)
C7—N1—C2—S1	−3.7 (6)	N1—C7—C8'—C9'	176.16 (17)
C6—N1—C2—S1	−179.4 (4)	C7—C8'—C9'—C10	0.00 (3)
N1—C2—N3—C4	−4.8 (6)	C8—C9—C10—C11'	19.9 (5)
S1—C2—N3—C4	179.1 (3)	C8—C9—C10—C9'	−81 (3)
C2—N3—C4—C5	5.2 (6)	C8—C9—C10—C11	−0.03 (7)
C2—N3—C4—C13	−179.3 (4)	C8'—C9'—C10—C9	90 (3)
N3—C4—C5—C14	178.7 (4)	C8'—C9'—C10—C11'	0.01 (7)
C13—C4—C5—C14	4.0 (7)	C8'—C9'—C10—C11	−19.1 (5)
N3—C4—C5—C6	−5.1 (6)	C4—C5—C14—O1	178.2 (5)
C13—C4—C5—C6	−179.8 (5)	C6—C5—C14—O1	1.8 (7)
C2—N1—C6—C5	−4.9 (8)	C4—C5—C14—C15	−2.7 (8)
C7—N1—C6—C5	179.2 (4)	C6—C5—C14—C15	−179.2 (5)
C4—C5—C6—N1	4.8 (7)	C9—C10—C11—C12	0.06 (9)
C14—C5—C6—N1	−178.4 (4)	C11'—C10—C11—C12	−91 (2)
C2—N1—C7—C8	85.5 (4)	C9'—C10—C11—C12	18.5 (5)
C6—N1—C7—C8	−98.5 (5)	C10—C11—C12—C7	−0.06 (8)
C2—N1—C7—C12'	−79.0 (5)	C8—C7—C12—C11	0.04 (7)
C6—N1—C7—C12'	97.1 (5)	C12'—C7—C12—C11	78 (3)
C2—N1—C7—C8'	104.8 (5)	C8'—C7—C12—C11	−19.5 (5)
C6—N1—C7—C8'	−79.2 (6)	N1—C7—C12—C11	−177.66 (16)
C2—N1—C7—C12	−96.8 (4)	C9—C10—C11'—C12'	−19.3 (5)
C6—N1—C7—C12	79.2 (5)	C9'—C10—C11'—C12'	−0.02 (8)
C12'—C7—C8—C9	−17.9 (5)	C11—C10—C11'—C12'	80 (2)
C8'—C7—C8—C9	92 (2)	C8—C7—C12'—C11'	18.5 (5)
C12—C7—C8—C9	−0.01 (3)	C8'—C7—C12'—C11'	−0.03 (7)
N1—C7—C8—C9	177.52 (17)	C12—C7—C12'—C11'	−93 (3)
C7—C8—C9—C10	0.01 (3)	N1—C7—C12'—C11'	−176.23 (17)
C8—C7—C8'—C9'	−80 (2)	C10—C11'—C12'—C7	0.04 (8)

### Hydrogen-bond geometry (Å, °)

**Table:**

*D*—H···*A*	*D*—H	H···*A*	*D*···*A*	*D*—H···*A*
N3—H3N···O1^i^	0.88	2.05	2.920 (2)	168

Symmetry codes: (i) *x*, *y*+1, *z*.
